# An updated view on the centrosome as a cell cycle regulator

**DOI:** 10.1186/s13008-022-00077-0

**Published:** 2022-02-14

**Authors:** Muyang Lin, Shuang Shuang Xie, Kuan Yoow Chan

**Affiliations:** 1 Department of Breast Surgery, The Second Affiliated Hospital of Zhejiang University School of Medicine, Zhejiang University, Hangzhou, 310029 People’s Republic of China; 2 Zhejiang University-University of Edinburgh Institute, Zhejiang University School of Medicine, Zhejiang University, Haining, 314400 People’s Republic of China

**Keywords:** Centrosome, SPB, Cell cycle, Cancer

## Abstract

The centrosome is a multifunctional organelle that is known primarily for its microtubule organising function. Centrosomal defects caused by changes in centrosomal structure or number have been associated with human diseases ranging from congenital defects to cancer. We are only beginning to appreciate how the non-microtubule organising roles of the centrosome are related to these clinical conditions. In this review, we will discuss the historical evidence that led to the proposal that the centrosome participates in cell cycle regulation. We then summarize the body of work that describes the involvement of the mammalian centrosome in triggering cell cycle progression and checkpoint signalling. Then we will highlight work from the fission yeast model organism, revealing the molecular details that explain how the spindle pole body (SPB, the yeast functional equivalent of the centrosome), participates in these cell cycle transitions. Importantly, we will discuss some of the emerging questions from recent discoveries related to the role of the centrosome as a cell cycle regulator.

## Centrosome abnormalities in cancer

The centrosome is a non-membrane bound organelle that acts as the main microtubule organising centre in the cell. In animal cells, it consists of a pair of microtubule-based cylinders called centrioles, which are embedded in the pericentriolar matrix (PCM) of proteins. The structure of the centrosome is highly ordered and its biogenesis is intimately linked to the cell cycle [1–3]. Centrosomes are involved in many cellular processes including mitotic spindle assembly, cell cycle progression, neurogenesis, cell polarity and migration [4]. As a result, centrosomal abnormalities can lead to a wide range of human diseases including cancer [5].

The concept that centrosomal abnormalities are associated with tumour progression was first postulated by Theodor Boveri more than 100 years ago [6]. Extensive clinical studies on multiple different cancer types supported Boveri’s hypothesis that centrosome abnormalities, in particular, centrosome amplification is associated with advanced tumours [7–11]. This conclusion is further reinforced by a recent systematic survey on centrosome abnormalities on the NCI-60 panel of human cancer cell lines where they show that centriole amplification is common and is correlated with aggressive breast and colon cancer cell lines [12]. Due to this strong association, a pan-cancer transcriptome analysis was conducted to generate a centrosome amplification signature, called CA20 [13]. This signature demonstrated prognostic value in independent breast cancer datasets, showing a strong correlation between a poor clinical outcome with a high CA20 value [13, 14].

While centrosome amplification is strongly associated with tumorigenesis, it was not clear if centrosomal amplification is an indicator of tumorigenesis or a contributor to tumorigenesis. A causative relationship could not be established until 2008, when Renata Basto while working at Jordan Raff’s lab, showed that inducing centrosome amplification in fruit flies resulted in the formation of tumour masses [15]. This was achieved by overexpressing the drosophila Polo-like kinase 4 (Plk4) homologue, SAK, which plays a key role in initiating centriole biogenesis [16–18]. This approach was later exploited in animal mouse models but produced variable results. Initial attempts to overexpress Plk4 failed to induce accelerated development of tumours, despite observing supernumerary centrosomes and a high incidence of aneuploidy in affected tissues [19–21]. This was partially due to the tumour suppressive effects of p53, as transient overexpression of Plk4 did induce tumour development in p53-deficient epidermis cells [22]. Another contributing factor was chronic overexpression of Plk4 resulted in cells with a large number of centrosomes, causing gross chromosome mis-segregation errors which were detrimental to cell viability. A more modest increase in Plk4 expression and centrosome number facilitated spontaneous tumour formation in mice and recapitulated features of chromosomal instability in human tumours [23].

It is important to note that impairing centrosome structure can also promote tumorigenesis. This was initially speculated in Gonzalez’s lab where they observed increased tumour growth potential in *Drosophila* tissues with mutations impairing centriole duplication [24]. Consistent with their conclusion, drug-induced inhibition of centriole duplication in non-transformed prostate epithelial cells resulted in the formation of malignant prostate tumours in animal models [25]. Furthermore, in a recent cancer genome analysis, mutations on several centrosomal components have been identified as tumour drivers [26]. Therefore, it is more appropriate to use the term centrosome abnormalities rather than centrosome amplification since structural changes or numerical changes to normal centrosomal morphology can induce tumorigenesis.

So, how does centrosome abnormality induce tumorigenesis? The role of centrosomes in establishing a bipolar spindle and mediating proper chromosome segregation is well established [27–29]. However, there is accumulating evidence that centrosomal abnormalities can induce tumorigenesis independent of their microtubule organising function. Work by Susana Godinho and David Pellman has established that centrosome amplification itself can promote invasive phenotypes in mammary epithelial cells when grown in a three-dimensional culture system [30]. This invasive property was attributed to increased oxidative stress levels within the cells, resulting in the secretion factors such as IL-8 that alters the cellular microenvironment, promoting cell invasion [31, 32]. These recent reports tell us that we do not fully comprehend the different cellular functions the centrosome has and how these functions contribute to tumorigenesis in cells experiencing centrosomal abnormalities.

In this review, we are focusing on the role of the centrosome as a cell cycle signalling hub. This is not a new concept as it has been introduced on several occasions in the past [33, 34]. Here, we aim to provide an updated view on the evolution of this idea due to recent discoveries. First, we review the historical evidence that led to the proposal that centrosomes participate in cell cycle regulation. We will then summarize the recent body of work that shows how the centrosome participates in the regulation of cell cycle signalling. Finally, we will discuss some of the major questions that arise from recent discoveries.

## The centrosome is involved in cell cycle progression

The idea that the centrosome may play a role in regulating the cell cycle was supported by the initial observation that microsurgical removal of centrosomes in BSC-1 African green monkey karyoplasts resulted in cell cycle arrest [35, 36]. Follow up studies involving laser ablation of centrosomes or RNAi mediated depletion of centrosomal components showed that cells without core centrosomal structures failed to progress to S phase, supporting the conclusion that centrosomes participate in cell cycle progression [36–38]. Apart from inhibiting cell cycle progression, it was observed that the depletion of centrosomal components also resulted in cytokinesis defects [38–40]. This indicates that the centrosome is involved at multiple points of the cell cycle.

**Fig. 1:**
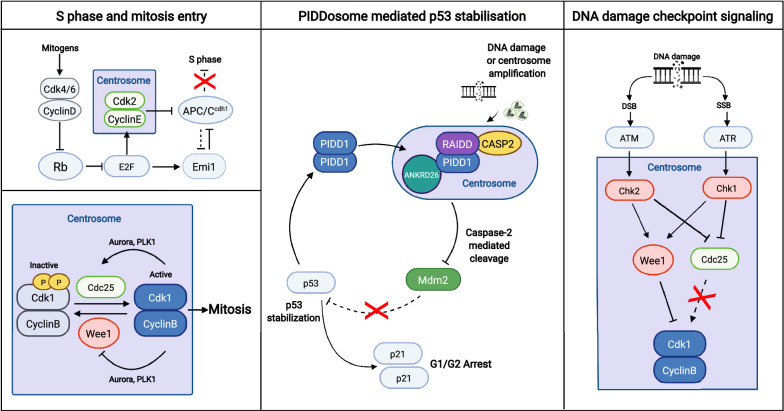
Regulation of cell cycle progression from the centrosome. The centrosome plays an important role as a signalling hub during the cell cycle. It facilitates the G1-S transition (top left) and the G2-M transition (bottom left) by recruiting key cell cycle players to the centrosome. The centrosome is also linked to the checkpoint signalling by anchoring the DNA damage checkpoint proteins (right) and the PIDDosome (centre). In the presence of cellular stress such as centrosome amplification and DNA damage, the centrosome promotes the checkpoint responses, which directly or indirectly inhibit the Cdk-Cyclin complexes and arrest the cell cycle

Cell cycle transitions are driven primarily by the activation of a family of kinases called the Cyclin-dependent kinases (Cdks). In G1, entry into S-phase in mammalian systems depends on the activation of Cdk4/6 complexes by mitogens, initiating a signalling cascade that results in the inactivation of the Retinoblastoma (Rb) protein and the activation of Cdk2 complexes (Fig. 1) [41–47]. In 1999, it was reported that Cdk2-Cyclin E was associated with the centrosome during interphase [48]. In 2004, the domain responsible for targeting Cyclin E to the centrosome was identified [49]. This domain which the authors called centrosome localisation signal (CLS) was found to be conserved between Cyclin A and Cyclin E [49, 50]. More importantly, systematic experimentation using Cyclin E mutants that lack these CLS motifs showed that Cyclin E recruitment to the centrosome is required for S-phase entry [49, 51].

In the G2 phase, the activation of Cdk1-Cyclin B and its translocation from the cytoplasm into the nucleus drives entry into mitosis [52–57]. The discovery that the cell cycle regulator Cdk1 is recruited to the centrosome in a cell-cycle dependent manner in the late 1980s / early 1990s, fuelled the speculation that the presence of Cdk1 at the centrosome is required for the activation of Cdk1-Cyclin B and mitotic entry [35, 58–60]. Consistent with this view, active Cdk1-Cyclin B is first observed on the centrosome in G2 before spreading into the nucleus before nuclear envelop breakdown [61]. Cdk1-Cyclin B activity is restrained in interphase through inhibitory phosphorylation in the active site of Cdk1 by Wee1 kinases (Fig. 1) [62–65]. These phosphates are removed by Cdc25 phosphatase to drive cells into division [66–69]. Both Cdc25 and Wee1 have centrosomal fractions in interphase indicating that the core components required for the regulation of Cdk1-Cyclin B activation are present at the centrosome [70].

The activation of Cdk1-Cyclin B is facilitated by mitotic kinases, Aurora A and Plk1 [71–73]. Both Aurora A and Plk1 are recruited to the centrosome in G2, and activation of these two kinases during the G2 phase plays an important role in promoting timely mitotic entry [71, 73–76]. The recruitment of Aurora A in G2 to the centrosome is mediated by the PCM component Cep192 [77]. Centrosomal recruitment of Aurora A in G2 results in its self-activation, which in turn activates Plk1 [72, 76–78]. Plk1 then simultaneously activates Cdc25 and inhibits Wee1, triggering the transition into mitosis [70, 74, 79–82]. These lines of evidence, coupled with the observation of activation waves emanating from the centrosome throughout the cytoplasm in Xenopus eggs after fertilisation, support the idea that the centrosome acts as a staging area, coordinating cellular signalling to trigger mitosis [83].

It is worth noting while all these cell cycle regulatory proteins have centrosomal fractions in G2 before mitosis (Table 1), non-centrosomal locations have been reported on several of these cell cycle regulatory proteins as well. For example, in the mammalian system, Plk1 has a dynamic distribution pattern and localises to kinetochores in prometaphase [84], where it is crucial for ensuring proper microtubule attachment to kinetochores and the subsequent silencing of the spindle assembly checkpoint [85–87]. It is not clear how much these non-centrosomal localisations contribute to the induction of mitotic entry or if the signalling events on the centrosome in G2 is a pre-requisite for setting up the relocalisation of these proteins to the nucleus before nuclear envelop breakdown. As we lack direct evidence implicating the centrosomal activation in some of these cell cycle regulators in triggering cell cycle transitions (Table 1), it would be of interest to determine their contributions experimentally.

**Table 1:** Summary of experimental evidence implicating the activation of cell cycle regulators on the yeast SPB or metazoan centrosome in regulating cell cycle transitions

Protein/protein complex	Experimental evidence
Aurora A	Centrosomal localisation observed in human cells [71] Experimental evidence supporting a role in G2/M transition [71] Localised activation at the centrosome suggested from RNAi experiments in *C. elegans* [76]
Cdc25	Centrosomal localisation in interphase observed in human cells [70] Localised activation at the centrosome in triggering G2/M transition suggested in human cells from siRNA experiments [70, 152]
Cdk 1-Cyclin B	SPB localisation observed in fission yeast cells [60, 123] Experimental evidence demonstrating the localised activation at the SPB promotes G2/M transition in fission yeast [122, 123] Centrosomal localisation in G2 observed in human cells [56, 58] Activation of the complex at the centrosome was observed in G2 before spreading into the nucleus before mitosis in human cells [61]
Cdk 2- Cyclin E	Centrosomal localisation in interphase observed in Xenopus [48] Experimental demonstration of localised activation at the centrosome promotes G1/S transition in human cells [49–51]
Chk1	Centrosomal localisation in interphase observed in human cells [70, 99] Centrosomal localisation is impaired when PCM genes are mutated/disrupted suggesting a role in G2/M cell cycle arrest in human cells [92, 97, 98] Experimental evidence demonstrating localised activation at the centrosome results in cell cycle arrest in human cells [99]
Chk2	SPB localisation observed in fission yeast [144] Experimental evidence demonstrating localised activation at the SPB supports mitotic entry in fission yeast [144] Localisation of protein in the centrosome observed in human cells [153–155]
PIDDosome	Centrosomal localisation observed in human cells [104–106] Localised activation at the centrosome is required for the stabilisation of the p53 [104, 105]
Plk1	SPB localisation observed in yeast cells [122, 128, 156] Localised activation at the SPB induced G2/M transition in fission yeast cells [122] The cellular kinase activity of Plk1 influenced localized recruitment of PP1 on the yeast SPB [128, 129] Centrosomal localisation observed in human cells [75, 155] Localised activation regulating G2/M transition from the centrosome suggested from drug inhibition and RNAi experiments in human cells [73–75] Localised activation at the centrosome promoting mitotic entry was proposed from RNAi experiments in *C. elegans* [76]
Wee1	SPB localisation in interphase observed in yeast cells, cell cycle regulatory role proposed based on changes in distribution during cell cycle progression [157, 158] Centrosomal localisation in interphase observed in human cells [70, 159]

## Checkpoint signalling at the centrosome

Cell cycle checkpoints are essential for maintaining genomic integrity in proliferating cells. Following DNA damage, the cell must detect sites of DNA damage, and either transiently block cell cycle progression, or exit the cell cycle. The DNA damage response (DDR) signalling network is cell cycle-dependent [88, 89]. In G2, both ataxia telangiectasia mutated (ATM) and ataxia telangiectasia and Rad3-related (ATR) signalling pathways are activated upon exposure to DNA damage (Fig. 1) [88, 90]. Chk1 and Chk2, which are downstream of ATM and ATR, play a key role in arresting the cell from progressing into mitosis through the inhibition of Cdk1-Cyclin B.

The centrosome is linked to the DNA damage checkpoint response as mutations on several PCM components (PCNT, MCPH1 and CDK5RAP2) have been shown to impair checkpoint mediated cell cycle arrest [91–97]. Cells defective in these centrosomal components are unable to respond to ATR signalling, failing to arrest its cell cycle at the G2/M boundary upon exposure to DNA damage [92, 96, 97]. The loss of G2/M checkpoint arrest was attributed to the failure to recruit Chk1 to the centrosomes [92, 97, 98]. Chk1 recruitment to the centrosome is cell-cycle dependent and is present in interphase cells but absent during mitosis [99]. Chemical inhibition of Chk1 causes premature centrosome separation, a result of accelerated activation of centrosome associated Cdk1 by Cdc25B [99]. More importantly, ectopic expression of Chk1 fusion proteins artificially targeted to the centrosome induced polyploidization as cells fail to enter mitosis, while kinase-dead controls did not [99]. Therefore, multiple PCM components are involved in the anchoring of the DNA damage checkpoint protein Chk1 and its recruitment to the centrosome is required to regulate the activation of Cdk1-Cyclin B at the centrosome.

The activation of the DNA damage response pathway also triggers p53 signalling. The canonical pathway that regulates p53 signalling is through a p53-specific E3 ubiquitin ligase known as Mdm2 [100, 101]. In the presence of DNA damage and other cellular stress, Mdm2 is cleaved, leading to the accumulation of p53 and the promotion of cell cycle arrest or cellular death [102, 103]. Recently, it was found that the centrosome can participate in the activation of p53 signalling through the PIDDosome which is anchored to the centriolar distal appendages (Fig. 1) [104–106]. The PIDDosome is a protein complex composed of PIDD1, Caspase-2 and RAIDD. It is best known for its function as an inducer of apoptosis [107]. In 2020, two back to back publications showed that in response to centrosome amplification, the centriolar distal appendage protein ANKRD26 recruits PIDD1 to the centrosome [104, 105]. They both show that the recruitment of the PIDDosome to the centrosome was required for Caspase-2 mediated cleavage of Mdm2 in response to centrosome amplification [104, 105]. Another important observation was that DNA damage-induced cleavage of Mdm2 appears to require ANKRD26 [105]. This suggests that the centrosome may be involved in other PIDDosome dependent p53 stabilisation responses [105, 107, 108].

### What happens when centrioles are lost?

In 2015, a selective inhibitor for Polo-like kinase 4 (Plk4) called centrinone was developed allowing specific inhibition of centriole duplication [109]. Prolonged exposure to centrinone caused the dilution of centrioles inside proliferating cells over time, leading to the formation of centriole-less daughter cells. The loss of centrioles impaired regular centrosome assembly leading to the accumulation of cells that lack a detectable Cep192 or γ-tubulin foci in interphase cells [109]. Following centrinone treatment, cell proliferation continued in cancer-derived HeLa cells while the non-transformed cell line RPE1 was arrested in a p53 dependent manner [109]. Follow up studies validated these initial observations and showed that the p53 mediated arrest in RPE1 was due to a mitotic surveillance pathway that was activated upon exposure to an extended mitotic duration [110–112]. These results, coupled with the observation that microsurgery or laser-induced ablation of centrosomes did not ubiquitously cause G1 arrest after centrosome removal [37], indicate that cell cycle progression is not inherently tied to the presence of an intact centrosome.

So, what happens with the PCM components in cells that experience centriole loss? Fluorescent live-cell imaging on centrinone treated RPE1 cells show that despite the loss of centrioles, endogenously tagged Cep192 will form a discrete foci to facilitate bipolar spindle assembly, as the cell progresses from G2 to early prophase [113, 114]. This indicates that in the absence of centrioles, at least some of the PCM components are dispersed within the cell in interphase. The loss of centrioles in RPE1 cells also caused the relocalisation of several PCM components (AKAP9, CDK5Rap2 and PCNT) to the Golgi Apparatus (GA) [115]. The relocalisation of these centrosomal components to the GA lead to the association of γ-tubulin to the GA, increasing the microtubule nucleating capacity of the Golgi [115]. The relocalisation of centrosomal components to other organelles do occur naturally in mammalian systems. Postnatal cardiomyocytes undergo a developmental process that results in the loss of centrioles as they become terminally differentiated [116–118]. This leads to centrosome disassembly and the relocalisation of PCM components to the perinuclear membrane [116–118]. As a result, γ-tubulin becomes associated with the perinuclear membrane transferring the microtubule organising function from the centrosome to the perinuclear membrane [116]. These observations show that the loss of centrioles causes the relocalisation of centrosomal proteins to other cellular compartments, transferring the microtubule organising function of the centrosome to the compartments they associate with. It is highly likely that the centrosomal function as a biological concentrator to facilitate Cdk activation and drive cell cycle transitions would be transferred in a similar fashion.

If cell cycle transitions can still occur in the absence of a concentrated foci of PCM components surrounding a centriole core, what would be the biological importance for a canonical centrosome structure? The answer to this question perhaps lies with the role of the centrosome in regulating the cell cycle transitions during checkpoint signalling. We already know that anchoring some signalling complexes to the centrosome is required for their biological activation. As discussed earlier, the recruitment of the PIDDsome to the centrosome is necessary for the triggering of Caspase-2 mediated stabilisation of p53 [104–106, 108]. Disruption of this localisation by centriole depletion blocks PIDDsome mediated p53 stabilisation [104, 105]. It is not difficult to imagine that other centrosomal signalling events could be disrupted when centrioles are absent. For example, the recruitment of Chk1 to the centrosome is required for arresting cells at the G2/M boundary when there is DNA damage [92, 97, 98]. This necessity is due to the requirement for Chk1 to downregulate Cdk1-Cyclin B activation on the centrosome [99]. Consequently, if the concentration of PCM components around the centrioles is disrupted, the proximity of these cell cycle regulatory proteins could be impaired. This would lead to the failure of checkpoint signalling within the cell to arrest cell cycle progression as they are unable to efficiently influence the activation of Cdks.

### Signalling insights from fission yeast

Much of our conceptual understanding of how the centrosome regulates the cell cycle is derived from work on model organisms, in particular, the fission yeast *Schizosaccharomyces pombe*. The fission yeast is a unicellular rod-shaped organism that grows by tip extension. In an unperturbed cell cycle, the length of the fission yeast cell is intricately tied to its cell cycle status, giving it a convenient physical characteristic to study cell cycle regulation [119–121]. This feature in combination with a haploid and a highly malleable genome allows for precise study of the cell cycle regulatory function of the yeast functional equivalent of the centrosome, called the spindle pole body (SPB). Like mammalian cells, the yeast version of Cdk1-Cyclin B, called Cdc2-Cdc13 accumulate at the SPB before mitotic entry [58, 60]. To directly test if centrosomal activation of Cdk1-Cyclin B is responsible for triggering mitotic entry, members from Iain Hagan’s lab generated conditionally active Cdk1^Cdc2^ kinase and targeted small amounts of these proteins to the SPB, nuclear envelope, cell tips or centromeres [122]. We found that only active Cdk1^Cdc2^ targeted to the SPB resulted in a burst of mitotic cells, demonstrating the importance of the localised activation of Cdk1 at the centrosome in promoting the mitotic entry [122]. This conclusion was supported by recent studies from Paul Nurse’s group where they found that abolishing yeast Cyclin B^Cdc13^ binding to the SPB prevented entry into mitosis in fission yeast [123]. The recruitment of Cyclin B to the centrosome was mediated by a highly conserved hydrophobic patch and mutating this hydrophobic patch in human Cyclin B abolished its centrosomal recruitment in U2OS cells, indicating that this mechanism is conserved from yeast to man [123].

While it is clear that the activation of Cdks at the centrosome drives cell cycle transitions, how the centrosome does this is not well understood. Hints on how the centrosome functions as a cell cycle regulator may be found from the work done on a SPB scaffold protein Cut12. In 1990 in a genetic screen to identify novel cell cycle regulators resulted in the identification of a mutant suppressor gene called *stf1* which overcomes the loss of a key mitotic activator Cdc25 phosphatase [124]. The sequencing of the *stf1* mutant gene led to the discovery that it codes for a SPB component Cut12 [125]. Follow up studies on Cut12 lead to the conclusion that Cut12 is a pro-mitotic signalling scaffold as the conditional loss of the function mutant *cut12.1* is synthetically lethal when combined with the conditional loss of function mutant *cdc25.22* but suppressed by boosting Cdc25 phosphatase levels [125–127]. In 2013, the molecular mechanism of how a single point mutation on Cut12 allowed the suppression was identified [128]. The mutation G71V in Cut12 impaired the binding of protein phosphatase 1, PP1^Dis2^ to Cut12 [128]. The decreased PP1^Dis2^ affinity for Cut12 resulted in the hyperactivation of Plk1^Plo1^, allowing cells to enter mitosis in the absence of Cdc25 activity [129]. Previous work from Iain Hagan’s lab has established that the activation of Cdk1^Cdc2^ at the yeast SPB is responsible for the recruitment of Plk1^Plo1^ and this recruitment was transient, with a protein turnover half-life of 22 s [122]. Blocking PP1^Dis2^ binding to Cut12 extended the duration where Plk1^Plo1^ was recruited to the SPB before mitosis and coincided with the increase in total kinase activity of Plk1^Plo1^ within the cell [122, 128, 129]. These observations support the idea that the SPB is acting as a biological concentrator where localised Cdk activation can influence the global phosphorylation states of multiple signalling molecules simultaneously. Consistent with this view, blocking the recruitment of yeast CyclinB^Cdc13^ and thus the activation of Cdk1^Cdc2^ at the SPB significantly impairs the phosphorylation of Cdk substrates in the cytoplasm [123].

**Fig. 2:**
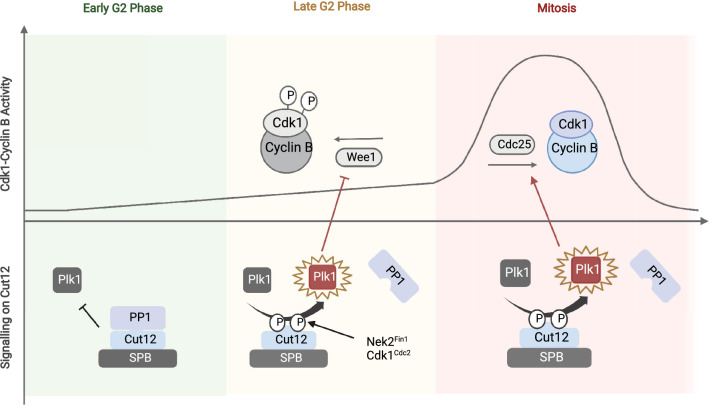
Localised activation of Plk1^Plo1^ at the yeast SPB reinforces the pro-mitotic signalling to drive cells into mitosis. The recruitment of PP1^Dis2^ to Cut12 negatively regulates the recruitment of Plk1^Plo1^ at the yeast spindle pole body (SPB). In G2, 30 min before mitosis, Nek2^Fin1^ and Cdk1^Cdc2^ activation at the centrosome expels PP1^Dis2^ from the SPB by phosphorylating the residues T75 T78 on Cut12. The disassociation of PP1^Dis2^ from the SPB allows the recruitment and activation of Plk1^Plo1^ at the SPB. The activation of Plk1^Plo1^ promote the inhibition of Wee1 and facilitates Cdc25 activation, reinforcing the commitment into mitosis by favouring the activation of Cdk1^Cdc2^

The interaction between the PP1^Dis2^ with Cut12 at the SPB serves an important biological function in the fission yeast cell. Within the PP1 binding motif on Cut12, there are two phosphorylation sites T75 and T78 [128]. T75 is a substrate for Cdk1^Cdc2^ and T78 is a substrate for Nek2^Fin1^ [128]. In an unperturbed cell cycle, T75 and T78 are phosphorylated in late G2 to expel PP1^Dis2^ from the SPB, which in turn allows the activation of Plk1^Plo1^ to promote commitment into mitosis (Fig. 2) [128]. The duration where Plk1^Plo1^ is recruited to the SPB before mitosis is dependent on exposure to environmental cues like cellular stress [122, 130]. Changes in Plk1^Plo1^ recruitment to the SPB, in turn, impact the timing of mitotic entry [122, 129, 130]. These observations suggest that the centrosome is regulating the timing of mitotic commitment by altering the balance between anti-mitotic and pro-mitotic signalling molecules at the centrosome.

Apart from regulating entry into mitosis, the yeast SPB also plays an integral role in regulating exit from mitosis. The events of mitotic exit and cytokinesis are regulated by a signalling network termed septation initiation network (SIN) in fission yeast and mitotic exit network (MEN) in budding yeast [131–135]. The Sid4-Cdc11 complex acts as a cytokinesis signalling hub on the fission yeast SPB by recruiting key SIN signalling components and regulators [136–139]. The activation of the SIN complex is closely linked to mitotic progression and is silenced when the spindle assembly checkpoint (SAC) is activated. An important component of the cytokinesis inhibition pathway is the yeast ubiquitin ligase Dma1, which is recruited to Sid4 when the SAC is activated [136, 140–142]. Deleting or blocking the recruitment of Dma1 to Sid4 would result in premature initiation of cytokinesis before mitosis is completed leading to anucleate or cut nucleus [140–142]. It is assumed that Sid4 and Cdc11 are cytokinesis specific scaffold proteins as mutations affecting either Sid4 or Cdc11 result in multinucleated cells because mitotic progression is unaffected but the triggering of cytokinesis was impaired [136, 139]. Some of the cytokinesis signalling components are conserved between yeast and man. For example, there are high levels of sequence conservation at the N-terminus of the human Centriolin gene when compared to their yeast counterparts Cdc11 and Nud1 [143]. This conservation likely means that Centriolin may be a functional homologue of Cdc11 and could act as a cytokinesis signalling hub in mammalian systems. This conclusion is supported by RNAi mediated knockdown of Centriolin which results in cytokinesis defects [143].

**Fig. 3:**
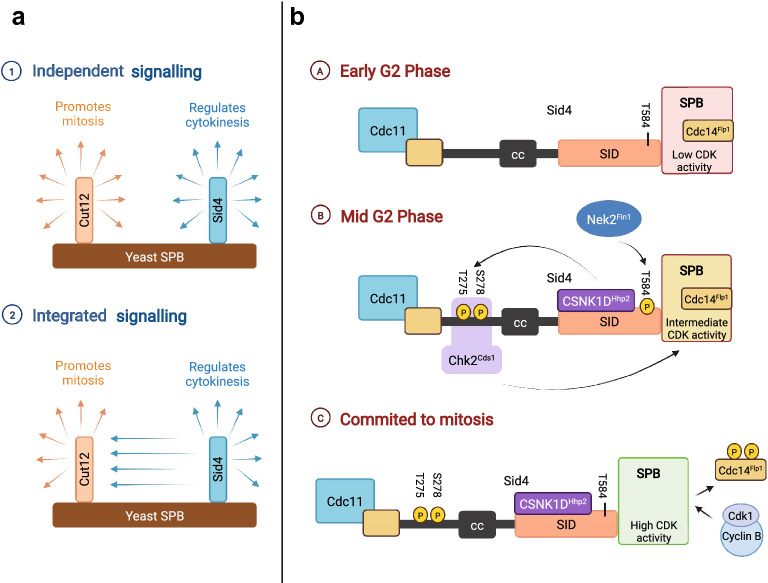
Crosstalk between two SPB scaffolds demonstrates the integration of signalling to promote cell cycle transitions. **a** A cartoon depicting the integration of signalling at the yeast SPB. It was previously thought that signalling scaffolds such as Cut12 and Sid4 work independently to promote cell cycle events and are functionally and temporally separated (top). Recent work shows that crosstalk between two functionally distinct signalling scaffolds occurs, working together to promote mitotic entry (bottom). **b** Model of the centrosomal signalling on Sid4 triggering mitosis. In mid G2, Nek2^Fin1^ recruits CSNK1D^Hhp2^ by phosphorylating Sid4 at residue T584. Recruitment of CSNK1D^Hhp2^ results in the phosphorylation of residue T275 and S278. This recruits Chk2^Cds1^ that phosphorylates Cdc14^Flp1^, expelling the phosphatase from the SPB. The absence of Cdc14^Flp1^ allows Cdk1-Cyclin B to activate the SPB triggering mitotic entry (CC: coiled-coil domain; SID: Sid domain, conserved N-terminal domain of Sid4)

The general idea on how signalling occurs in the centrosome is that specialised signalling scaffolds such as the mitotic entry scaffold Cut12 is responsible for mitotic entry and the mitotic exit scaffold Sid4 is responsible for mitotic exit. Therefore, it was assumed that these signalling hubs on the centrosome is functionally separated and therefore operate independently. However, our recent work suggests that crosstalk between both signalling complexes occur and both Cut12 and Sid4 work together to promote mitotic entry (Fig. 3a) [144]. We found that phosphorylation of a single residue in the C-terminus of Sid4 by the Nek2^Fin1^ promotes the recruitment of CSNK1D^Hhp2^ to Sid4 (Fig. 3b). This recruitment results in the phosphorylation of T275 and S278 on Sid4 by CSNK1D^Hhp2^. Phosphorylated T275 S278 recruits Chk2^Cds1^ to expel the Cdc14^Flp1^ from the SPB [144]. Because Cdc14 family phosphatases target sites phosphorylated by Cdk1-Cyclin B [145], the expulsion of Flp1 reduces the level of local antagonism towards Cdk1-Cyclin B on the SPB, supporting mitotic activation of the defective SPB of *cut12.1* cells. Both CSNK1D^Hhp2^ and Chk2^Cds1^ are kinases that are associated with DNA damage signalling [146, 147]. The unexpected involvement of these DNA damage related kinases during mitotic entry is surprising as DNA damage related kinases are often associated with triggering checkpoint responses and delaying entry into mitosis. This demonstrates the dynamic nature of centrosomal signalling as the interaction between various centrosomal components can bring about unexpected outcomes. More importantly, the fact that signalling events on Sid4 could influence Cut12 activity during mitotic entry supports the idea that the centrosome is functioning as a signal integration hub (Fig. 3a), where signalling pathways are connected and transformed into a decision to trigger cell cycle transitions.

## Conclusion

The centrosome is a complex organelle that operates beyond its canonical role as a microtubule organising centre. While its role as a signalling platform mediating cell cycle transitions has been demonstrated in multiple eukaryotic systems, we still do not have a good grasp on the signalling events which bring about these transitions on mammalian systems. Furthermore, recent mapping of the mammalian centrosome interactome by proximity mediated biotinylation assay, has revealed a plethora of centrosomal interactors with a variety of biological functions including metabolism, protein synthesis, autophagy and inflammation [148, 149]. Many of these centrosomal interactions are biologically significant, as recent work has shown that some aspects of inflammasome and apoptosome signalling depend on its interaction with the centrosome [104–106, 150, 151]. As the cell cycle influences and respond to many biological events in the cell, it is tempting to speculate that the centrosome is an avenue for crosstalk to occur between these different signalling networks. Understanding how these interactions at the centrosome are translated to a decision to trigger cell cycle transitions will be important, as it will refine our understanding of centrosomal functions and provide a framework to reveal the molecular basis of how centrosomal defects induce human diseases.

## Data Availability

Not applicable.

## Abbreviations

PCM: Pericentriolar matrix
Cdk: Cyclin-dependent kinase
CLS: Centrosome localisation signal
DDR: DNA damage response
ATM: Ataxia telangiectasia mutated
ATR: Ataxia telangiectasia and Rad3-related
SPB: Spindle pole body
SIN: Septation initiation network
MEN: Mitotic exit network
SAC: Spindle assembly checkpoint
Plk1: Polo-like kinase 1
Plk4: Polo-like kinase 4
